# Stereotactic Body Radiotherapy for Inoperable Liver Tumors: Results of a Single Institutional Experience

**DOI:** 10.7759/cureus.935

**Published:** 2016-12-20

**Authors:** Hussam Hijazi, Marie-Pierre Campeau, David Roberge, David Donath, Real Lapointe, Franck Vandenbroucke-Menu, Daniel Taussky, Karim Boudam, Gabriel Chan, Alexis Bujold, Guila Delouya

**Affiliations:** 1 Department of Radiation Oncology, Centre hospitalier de l’Université de Montréal (CHUM); 2 King Abdulaziz University Hospital, Jeddah, Saudi Arabia; 3 Department of Radiation Oncology, Centre hospitalier de l'Université de Montréal (CHUM); 4 Department of Oncology, Division of Radiation Oncology, McGill University Health Center; 5 Department of Radiology, Radiation Oncology and Nuclear Medicine, University of Montreal; 6 Department of Radiation Oncology, Centre hospitalier de l'Université de Montréal (CHUM); 7 Department of Hepato-Biliary and Pancreatic Surgery, Centre hospitalier de l’Université de Montréal (CHUM); 8 Unit of Hepato-Biliary and Pancreatic Surgery, Centre hospitalier de l’Université de Montréal (CHUM); 9 Department of Hepato-Biliary and Pancreatic Surgery, Hôpital Maisonneuve-Rosemont; 10 Department of Radiation Oncology, Hôpital Maisonneuve-Rosemont

**Keywords:** sbrt, stereotactic body radiotherapy, liver cancer, hcc, metastatic liver tumors

## Abstract

**Objectives:**

Stereotactic body radiation therapy (SBRT) is an emerging treatment option for liver tumors unsuitable for ablation or surgery. We report our experience with SBRT in the treatment of liver tumors.

**Materials and methods:**

Patients with primary or secondary liver cancer were identified in our local SBRT database. Patients were included irrespective of prior liver-directed therapies. The primary endpoint of our review was in-field local control (LC). Secondary endpoints were progression-free survival (PFS), overall survival (OS), and toxicity.

**Results:**

From 2009 to 2015, a total of 71 liver lesions in 68 patients were treated with SBRT (three patients had two liver lesions treated). The median age was 71 years (27–89 years). Hepatocellular carcinoma (HCC) was the diagnosis in 23 patients (34%), with the grade of Child–Pugh A (52%), B (39%), or C (nine percent) cirrhosis. Six patients (nine percent) had intrahepatic cholangiocarcinoma (IHC). The remaining 39 patients (57%) had metastatic liver lesions. Colorectal adenocarcinoma was the most common primary tumor type (81%). The median size for HCC, IHC, and metastatic lesions was 5 cm (2–9 cm), 3.6 cm (2–4.9 cm), and 4 cm (1–8 cm), respectively. The median prescribed dose was 45 Gy (16–50 Gy).

Median follow-up was 11.5 months (1–45 months). Actuarial one-year in-field LC for HCC and metastatic lesions was 85% and 64% respectively (*p= 0.66*). At one year, the actuarial rate of new liver lesions was 40% and 26%, respectively, (p=*0.58*) for HCC and metastases. Only six patients with IHC were treated with SBRT in this study – in these patients, one-year LC was 78% with new liver lesions in 53%.

The SBRT treatments were well tolerated. The side effects included common criteria for adverse events (CTCAE) v4 grade 1 acute gastrointestinal toxicity in three patients, grade 3 nausea in one patient, and grade 3 acute dermatitis in another patient. Two patients had grade 5 toxicity. Radiation pneumonitis was observed in one patient two months post-SBRT treatment, and another patient was suspected to have had radio-induced liver disease (RILD) two months after SBRT. No late toxicity was seen.

**Conclusion:**

SBRT is a well-tolerated and effective alternative treatment option for selected patients with primary and metastatic liver tumors.

## Introduction

Primary liver cancer is the second-most common cause of cancer death worldwide, estimated to be responsible for nearly 745,000 deaths in 2012. The prognosis for liver cancer is poor (overall ratio of mortality to incidence of 0.95), and as such, the geographical patterns in incidence and mortality are similar. In addition, the liver is also one of the most common sites of metastases, particularly from colorectal carcinoma [1]. An estimated 50%–70% of patients diagnosed with colorectal cancer will suffer from liver metastases during the course of the disease.

Surgery is the main curative treatment for both primary and secondary liver cancer. However, surgical resection is feasible in only 20%–30% of cases [2-3].

There is a significant need for nonsurgical treatment modalities. Although systemic therapies are becoming more effective, long-term disease-free survival is unlikely without local therapy [4]. Without local treatment, patients are typically condemned to lifelong systemic treatment with the associated toxicities and costs. The most common liver-directed therapies are radiofrequency ablation (RFA), transarterial chemoembolization (TACE) and, more recently, SBRT. Radiofrequency ablation is an effective treatment for selected patients, but its application can be limited by factors such as lesion size and proximity to the gallbladder, main vessels, or diaphragm. TACE can provide a modest survival benefit for patients with HCC  as compared to the best supportive care [5]. TACE does have its own limitations – notably in cases with poor neovascularization, portal vein involvement, or compromised liver reserve.

Historically, conventional radiotherapy has had a limited role in the treatment of liver tumors because of the risk of RILD. In the last two decades, radiotherapy has experienced a technological revolution. Advances in radiotherapy have been seen in treatment planning, motion management, and image guidance, and have enabled precise partial liver irradiation to effect a significant reduction in the risk of RILD. Combined, these technological advances have enabled SBRT.

Recently, small phase I and II studies have tested the feasibility, safety, and toxicity of SBRT in the treatment of primary and metastatic liver cancer [4,6]. This growing body of evidence has led to the integration of SBRT in national cancer management guidelines [7].

In this study, we report on an initial experience with SBRT for primary and metastatic liver tumors.

## Materials and methods

This is a retrospective study conducted at our institution, Centre Hospitalier de l’Université de Montreal (CHUM), and approved by its ethics review board. Patients treated with SBRT for one or more liver lesions from 2009 to 2015 were identified retrospectively in a local SBRT registry. Informed consent was obtained from all the patients for this study.

### Patient characteristics

This study includes all patients diagnosed with primary or secondary liver cancer unsuitable for surgery or other local treatment, despite any previous therapy including surgical resection, chemotherapy, TACE, or RFA. Patients treated with conventional fractionated radiotherapy or palliative radiotherapy to the liver were excluded. Patient characteristics are outlined in Table 1.

**Table 1:** Patient characteristics

Factor	Number
Number of patients	68
Number of lesions	71
Age (range)	71 years (27–89)
Sex	
Male	41
Female	27
Diagnosis	
Primary	
Hepatocellular carcinoma	23
Intrahepatic cholangiocarcinoma	6
Secondary	
Colorectal adenocarcinoma	34
Other	8

### Treatment planning and delivery

**Figure 1:**
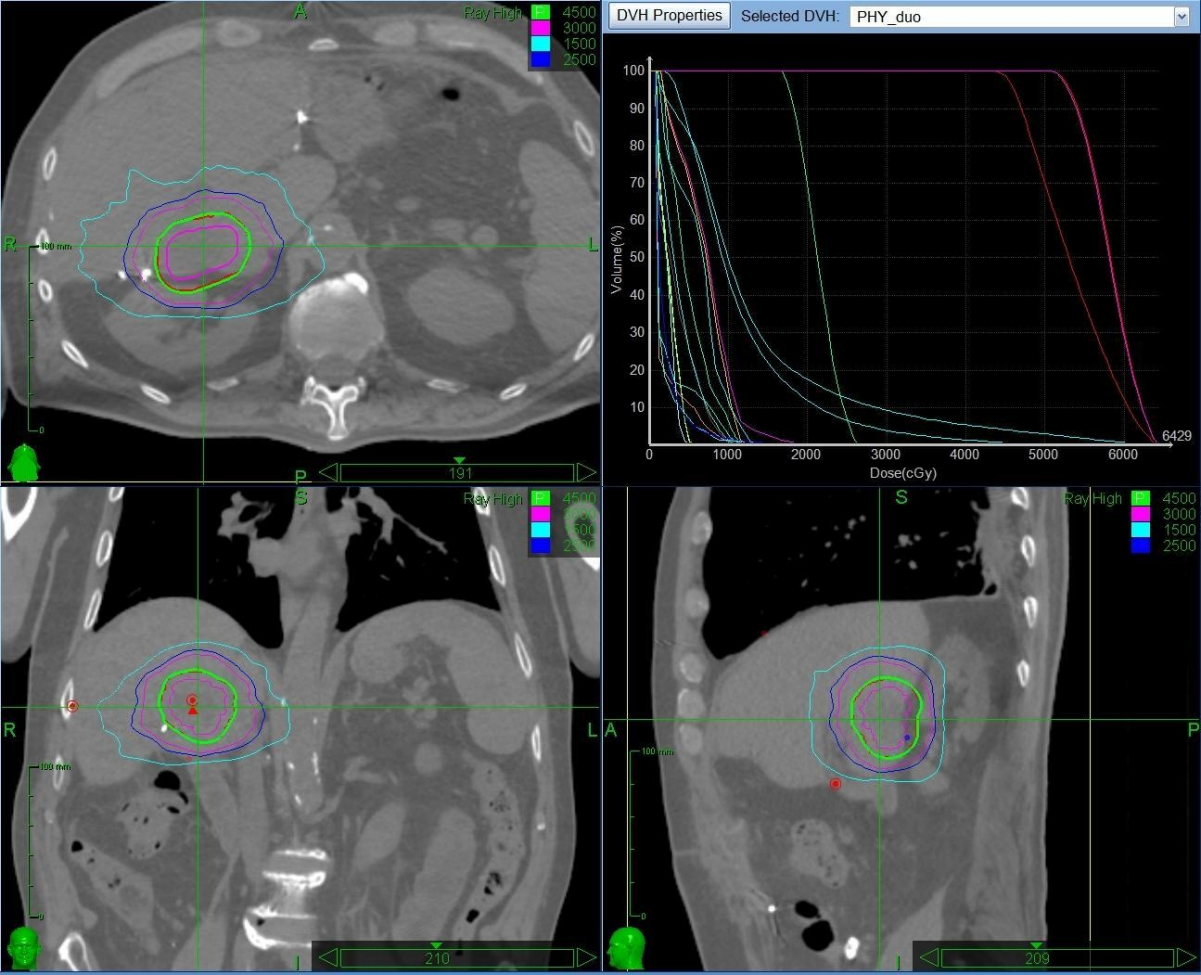
CyberKnife (Accuray, CA, USA) treatment planning

Three different treatment techniques are used in our department (fiducial-based tracking, internal target volume (ITV), or gated techniques). Irrespective of technique, all patients underwent a four-dimensional computed tomography scan (4DCT) – typically with a single phase intravenous (IV) contrast injection (arterial for primary tumors, venous for metastases). Patients also typically underwent a non-contrast T2-weighted planning magnetic resonance imaging (MRI) scan. Image coregistration helped define gross tumor volume (GTV). Patients treated with robotic stereotactic radiotherapy had tracking of implanted fiducial markers, surgical clips, or lipiodol from a prior TACE as seen in Figure 1.

**Figure 2:**
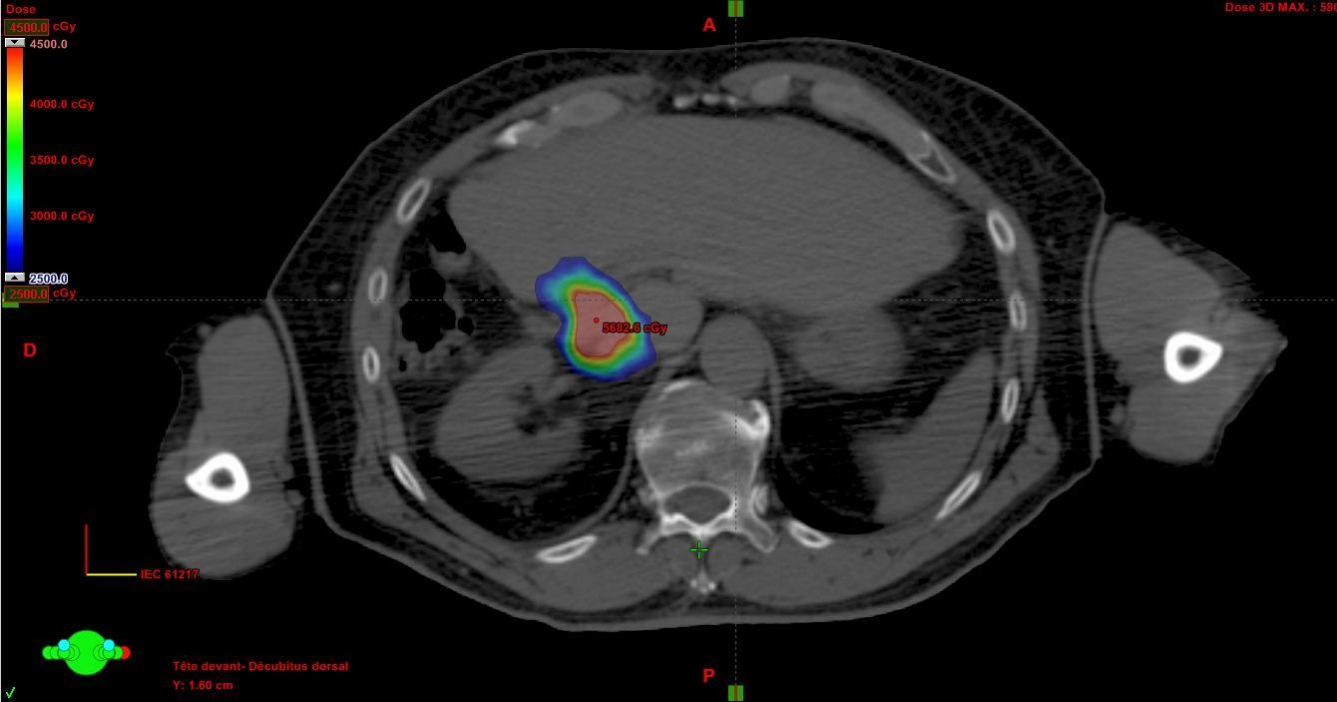
Volumetric intensity-modulated radiation therapy (IMRT) RapidArc (Varian Medical Systems, CA, USA) treatment planning IMRT: Intensity Modulated Radiation Therapy

In cases using the ITV technique, the 4D images were used to identify the target in various phases of the respiratory cycle as shown in Figure 2.

The gating technique was used for patients with a regular respiratory cycle and significant liver motion. Gating was most commonly performed in free-breathing. This method involves real-time monitoring and triggers the linear accelerator at a specific respiratory phase.

The most common prescription regimen was 45 Gy in three fractions, prescribed to the 70%–80% isodose volume. Treatment was delivered every other day over two weeks. Dosimetric constraints to organs at risk relied on Quantitative Analyses of Normal Tissue Effects in the Clinic (QUANTEC) recommendations for SBRT [8]. Liver optimization was based on sparing ≥ 700 cc of uninvolved hepatic parenchyma from receiving more than 17–21 Gy (depending on the fractionation used). For liver metastases, 45 Gy in three fractions has been chosen as the standard fractionation regimen according to previous experiences that showed a good LC with this regimen for tumors < 3 cm with acceptable toxicity [9-10].

### Follow-up

Patients were typically evaluated one-month posttreatment and then every three months for the first year and every six months thereafter. Follow-ups included clinical examination, liver imaging, and blood work. As our center is a regional reference center for SBRT, some patients were followed by their referring physician. Best efforts were deployed to obtain follow-up data from the referring physicians. Acute toxicity was defined as adverse events occurring within the first three months, posttreatment and late toxicities as adverse events occurring after three months. Common criteria for adverse events (CTCAE) v4.0 was used to grade adverse events. Response evaluation criteria in solid tumors (RECIST V1.1) were used to assess radiologic tumor response.

### Statistical analysis

LC and OS rates were calculated using Kaplan-Meier analysis. Log-rank testing was used to compare outcomes between the subsets of patients analyzed. Cox proportional hazards regression analysis was used for multivariate analysis. A p-value < 0.05 was considered significant. Data were analyzed with Statistical Package for the Social Sciences (SPSS) 17.0 for Windows (IBM, NY, USA). Local failure (in-field progression) was defined as occurring within the irradiated high dose volume.

## Results

Sixty-eight patients with a total of 71 liver lesions (three patients had two liver tumors) were identified. The median age was 71 (27–89 years). HCC occurred in 23 cirrhotic patients (32%) with Child–Pugh A (52%), B (39%), and C (nine percent). Six patients (nine percent) had IHC. The remaining 59% (39 patients) had metastatic liver lesions. Colorectal adenocarcinoma was the most common primary tumor type (34 patients). The median size for HCC, IHC, and metastatic lesions was 5 cm (2–9 cm), 3.6 cm (2–4.9 cm), and 4 cm (1–8 cm), respectively.

Tumor characteristics are shown in Table 2.

**Table 2:** Tumor characteristics *HCC*: hepatocellular carcinoma

Factors	Median	Range
Tumor size	4 cm	(1–9)
HCC	5 cm	(2–9)
Metastatic	4 cm	(1–8)
Prescribed dose	45 Gy	(16–50)
HCC	45 Gy	(16–50)
Metastatic	45 Gy	(24–50)
Dose/fraction	10 Gy	(5–15)
HCC	9 Gy	(5–15)
Metastatic	15 Gy	(5–15)
Number of fractions	5	(2–8)
HCC	5	(2–6)
Metastatic	3	(3–6)

**Figure 3:**
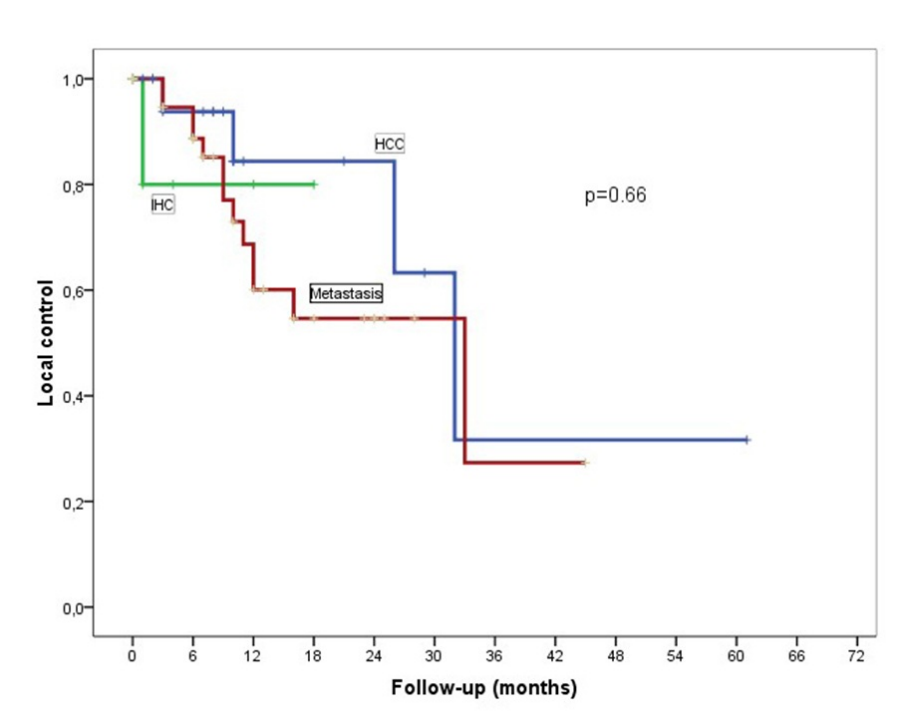
Kaplan–Meier curves showing LC for HCC, IHC, and metastatic patients

The median follow-up was 12 months from end of treatment to last follow-up or death (1–67 months). As illustrated in Figure 3, one-year LC rates for HCC and metastatic patients were 85% and 64%, respectively (p= 0.66).

The rate of one-year survival free of new liver lesions excluding local recurrence for HCC and metastatic patients was 60% and 74%, respectively (p= 0.58).

**Figure 4:**
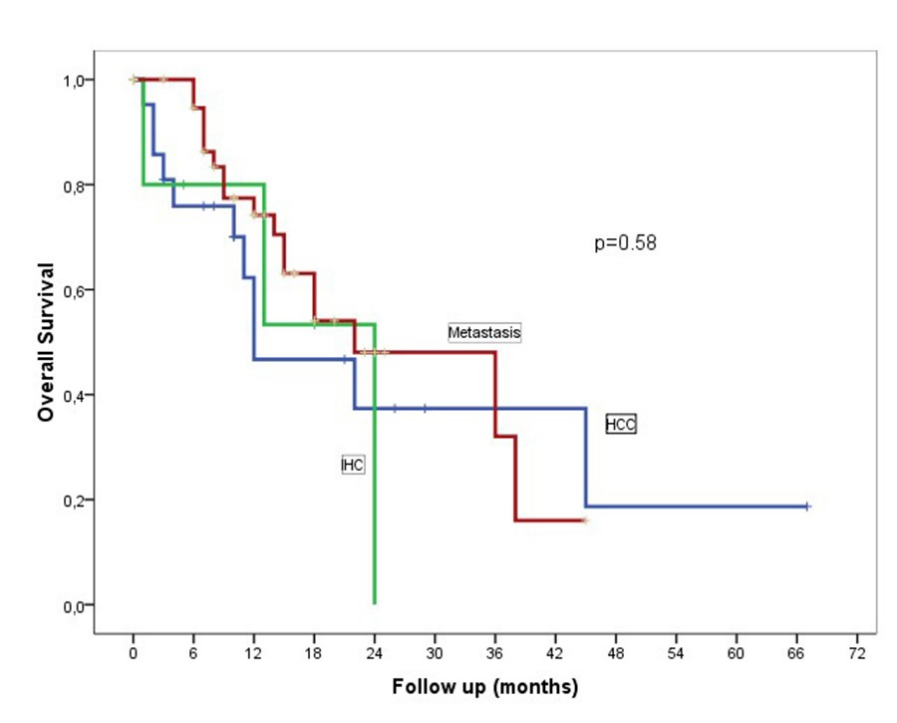
Kaplan–Meier curves showing OS for HCC, IHC, and metastatic patients

One-year disease-free survival for HCC and metastatic patients was 56% and 48%, respectively. Median OS was 11.5 and 13 months for HCC and metastatic liver cancer, respectively. Actuarial OS is plotted in Figure 4.

Only six patients with IHC were treated with SBRT in this study. LC at one year for IHC was 78% and new liver lesions occurred in 53%. The median OS for these six patients was 10 months. Three patients with IHC remain alive and free of progression at last follow-up – five, 13, and 18 months posttreatment. There was no difference on LC when analyzing for lesion size or dose prescribed.

Treatment was well tolerated with CTCAE v4.0 grade 1 acute gastrointestinal toxicity in three patients, grade 3 nausea in one patient, and grade 3 dermatitis in another. Two patients developed serious complications two months after radiation in the form of radiation pneumonitis and RILD which likely contributed to their deaths. Although the patient who had RILD was known to have class C cirrhosis diagnosed before the SBRT treatment, the progression in liver function and the clinical evolution of the patient met the criteria of RILD definition and was thus considered as RILD. Thus, radiation was implicated in the death of two out of five patients who died in the first 90 days following treatment (all cause 90-day mortality seven percent). No late toxicity was seen.

## Discussion

The aim of this retrospective study is to evaluate the safety and efficacy of SBRT in the treatment of liver tumors, as delivered at our institution.

In our series, actuarial one-year LC for HCC and metastatic liver cancer was 85% and 64%, respectively. Though our LC for HCC was within the range of what has been reported in the literature, in metastatic liver tumors, especially colorectal adenocarcinoma, our LC was lower but still within the range of published values [11-15]. The lower LC for metastatic lesions may in part be explained by the development of radio-resistance after protracted and varied chemotherapy.

Advances in technology such as tracking and adaptive gating may have an impact on reducing treatment toxicity as they enable tighter planning margins. Although the dose is prescribed to obtain the biological equivalent dose (BED) > 100 Gy10 [16], in selected cases, we did reduce the total dose and/or increase the fractionation in order to meet normal tissue constraints. For example, in cases of tumors located next to luminal structures (stomach or bowel), we used alternative regimens such as 45 Gy in five fractions. In other cases, we elected to accept a partial coverage of the planning target volume (PTV) in order to respect the surrounding normal tissue constraints.

The low incidence of symptomatic RILD observed in our series and in the published literature would suggest that it may be possible to evaluate escalated normal tissue tolerance levels [17]. In our clinic, advanced cirrhosis and general frailty remain relative but not absolute contraindications to therapy. Two patients in our series did die within 90 days of treatment with radiation as a probable cause. One, an 83-year-old patient, had probable pneumonitis following treatment of a 7 cm lesion in the hepatic dome. The other was a patient with Child–Pugh class C cirrhosis which rapidly deteriorated after SBRT. Overall, in our review, radiation was well tolerated with four patients (six percent) experiencing grade 3 or greater toxicity.

OS at one year for HCC and metastatic liver cancer was 63% and 77%, respectively. These results were also in accordance with what has been previously published [11-12,14-15,18]. Prior studies reported a wide range in the one-year OS rate (50%–100%) and this could be due to patient, treatment, and follow-up heterogeneity across studies [19-23]. The heterogeneous nature of the population included in this study with different tumor burden, pathology, liver function, and prior treatment makes the results difficult to compare [24].

The impact of SBRT on survival is not yet clear. Disease progression outside the treated lesion remains an issue. For HCC patients, adding a systemic treatment may help improve patient outcomes. A phase III randomized study comparing sorafenib versus SBRT followed by sorafenib will help clarify the role of this combined treatment in HCC [23].

SBRT must find a niche in relation to more established local treatments: surgical resection, liver transplantation, TACE, and RFA. Although RFA has been effective for small HCC lesions (< 3 cm) with a one-year recurrence-free survival rate of 74% [25], there may be a gain in combining RFA with SBRT for larger lesions for which RFA is less effective as a single modality [18]. A randomized study has already suggested a recurrence-free survival benefit for HCC patients treated with TACE-RFA when compared to RFA only [26]. Another retrospective study has suggested a survival benefit in patients treated with TACE followed by SBRT, over TACE alone for HCC tumors of > 3 cm [27]. Furthermore, liver SBRT may have a role in enabling HCC patients to remain on the list for liver transplantation. Case series have documented the feasibility of SBRT as a bridge to liver transplantation with minimal toxicity [28-29].

Our study has limitations in that it is a retrospective study with a relatively short follow-up period. The group is heterogeneous including primary and metastatic liver tumors treated with different dose regimens.

## Conclusions

The role of radiation therapy for primary and metastatic liver cancer has evolved over the years. Advances in technology have allowed escalation of tumor radiation while sparing normal liver parenchyma. Although prospective comparative evidence is lacking, SBRT appears to be a useful and safe means of providing LC of liver tumors in a select patient population.
